# Supplementary material for: Colchicine use in patients with COVID-19: A systematic review and meta-analysis
Source: PLoS One. 2021 Dec 28;16(12):e0261358. doi: 10.1371/journal.pone.0261358 (PMC8714120; doi:10.1371/journal.pone.0261358)
Supplement: S1 Appendix — (DOCX) [file pone.0261358.s002.docx]

**S1 Appendix.** Search Strategies

Database: Ovid MEDLINE(R) and Epub Ahead of Print, In-Process & Other Non-Indexed Citations, Daily and Versions(R) <1946 to March 24, 2021>

Search Strategy:

--------------------------------------------------------------------------------

1     (covid 19 or covid-19).mp. (95589)

2     "coronavirus disease 2019".mp. (18596)

3     SARS-CoV-2.mp. (37725)

4     or/1-3 (99157)

5     exp Colchicine/ (15163)

6     colchicine.mp. (20749)

7     colcrys.mp. (8)

8     mitigare.mp. (4)

9     gloperba.mp. (0)

10     or/5-9 (21710)

11     4 and 10 (90)

12     limit 11 to english language (88)

***************************

Database: Embase Classic+Embase <1947 to 2021 March 24>

Search Strategy:

--------------------------------------------------------------------------------

1     (covid 19 or covid-19).mp. (80912)

2     "coronavirus disease 2019".mp. (81124)

3     SARS-CoV-2.mp. (28874)

4     or/1-3 (93686)

5     exp Colchicine/ (35809)

6     colchicine.mp. (40067)

7     colcrys.mp. (73)

8     mitigare.mp. (2)

9     gloperba.mp. (0)

10     or/5-9 (40067)

11     4 and 10 (207)

12     limit 11 to english language (205)

***************************

Database: EBM Reviews - Cochrane Central Register of Controlled Trials <February 2021>

Search Strategy:

--------------------------------------------------------------------------------

1     (covid 19 or covid-19).mp. (3627)

2     "coronavirus disease 2019".mp. (707)

3     SARS-CoV-2.mp. (1379)

4     or/1-3 (3725)

5     exp Colchicine/ (337)

6     colchicine.mp. (930)

7     colcrys.mp. (2)

8     mitigare.mp. (0)

9     gloperba.mp. (0)

10     or/5-9 (931)

11     4 and 10 (40)

12     limit 11 to english language (7)

***************************

Database: medRxiv – The Preprint Server for Health Sciences <January 28, 2021>

--------------------------------------------------------------------------------

1     covid 19 AND (colchicine OR colcrys OR mitigare OR gloperba) (52)

***************************

Database: Research Square Preprint Platform <January 28, 2021>

--------------------------------------------------------------------------------

1     covid 19 AND (colchicine OR colcrys OR mitigare OR gloperba) (2)
