# Supplementary material for: Colchicine use in patients with COVID-19: A systematic review and meta-analysis
Source: PLoS One. 2021 Dec 28;16(12):e0261358. doi: 10.1371/journal.pone.0261358 (PMC8714120; doi:10.1371/journal.pone.0261358)
Supplement: S2 Appendix — (DOCX) [file pone.0261358.s003.docx]

**S2 Appendix.** PRISMA Flow Diagram

288 records excluded

129 full-text articles assessed for eligibility

417 records screened after duplicates removed (n=125)

540 records identified through database search

3 additional records identified

119 full-text articles excluded:

- Commentary article (n = 21)
- Review article (n = 51)
- Study protocol (n = 3)
- Case study/series (n=28)
- Guideline (n = 1)
- Does not report results by colchicine use in specific population (n = 15)

2 studies not reporting adjusted risk ratios were excluded

10 studies identified for potential quantitative synthesis

8 studies included in quantitative synthesis (meta-analysis)
