# Supplementary figures and images for: Colchicine use in patients with COVID-19: A systematic review and meta-analysis
Source: PLoS One. 2021 Dec 28;16(12):e0261358. doi: 10.1371/journal.pone.0261358 (PMC8714120; doi:10.1371/journal.pone.0261358)

**S3 Appendix.** Risk of Bias Assessment **3a** Observational Studies **3b** Randomized Controlled Trials

**a.**


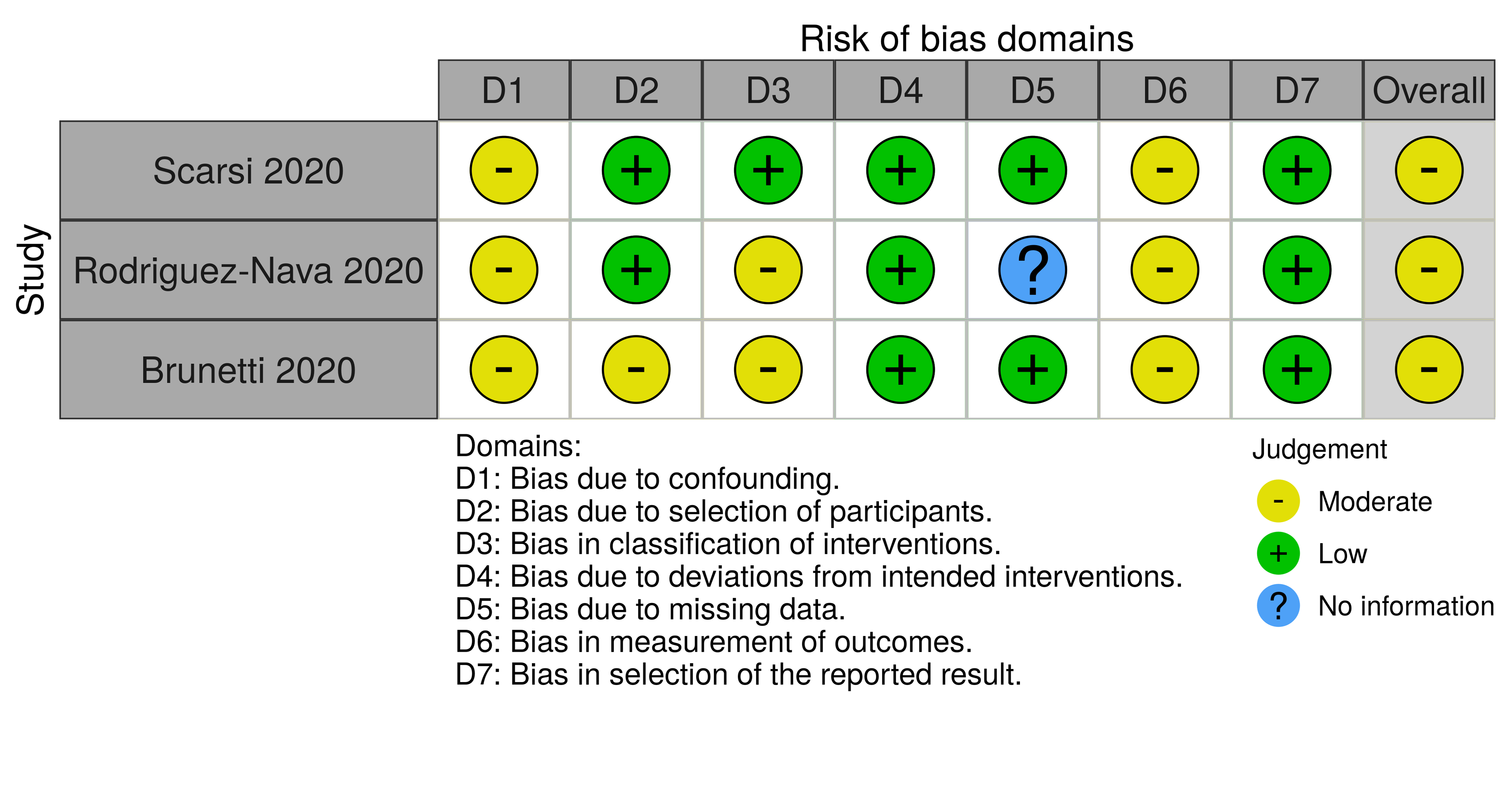


**b.**


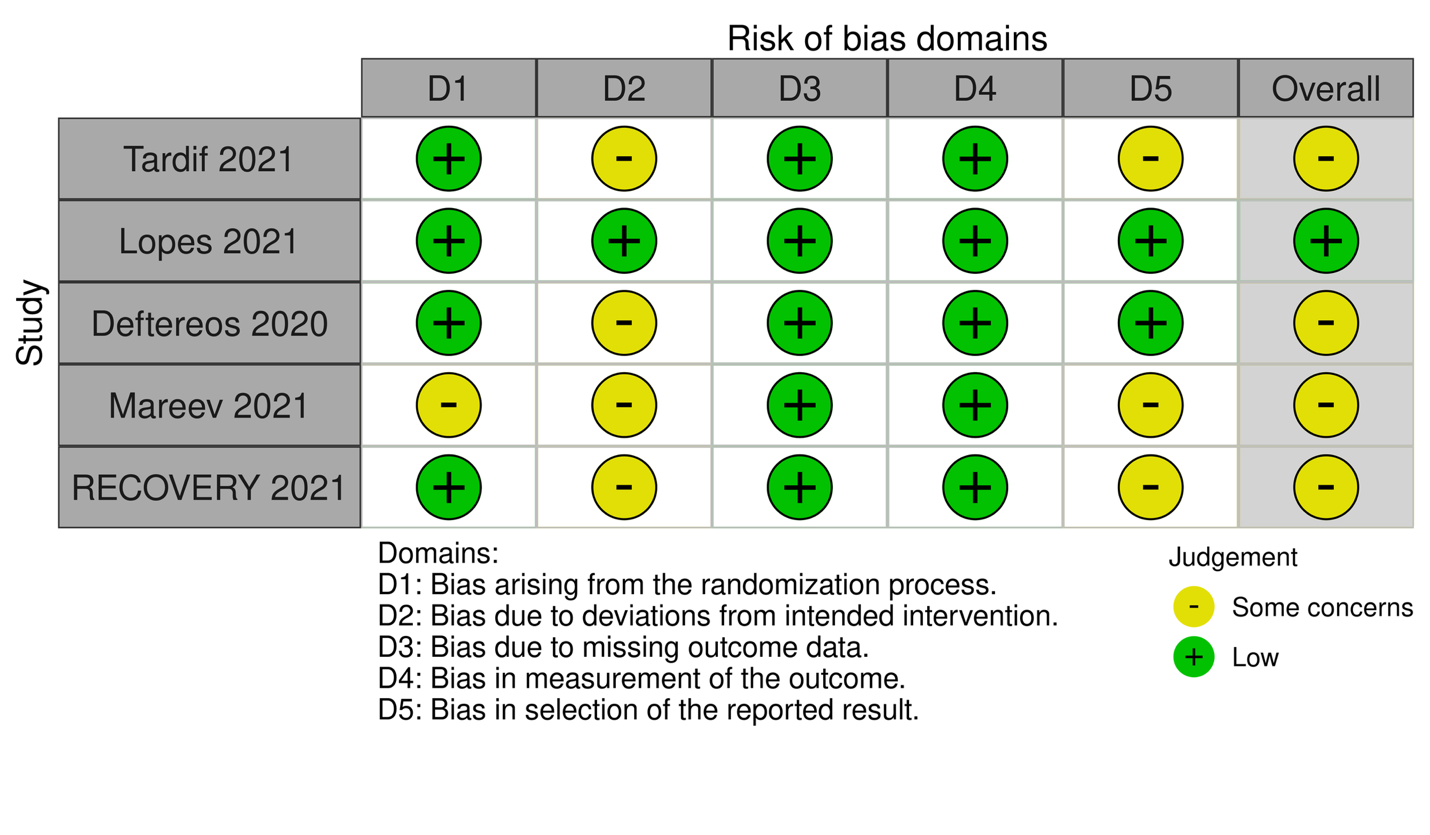

Supplement: S3 Appendix — (DOCX) [file pone.0261358.s004.docx]

**S4 Appendix.** Assessment for Publication Bias – Funnel Plot


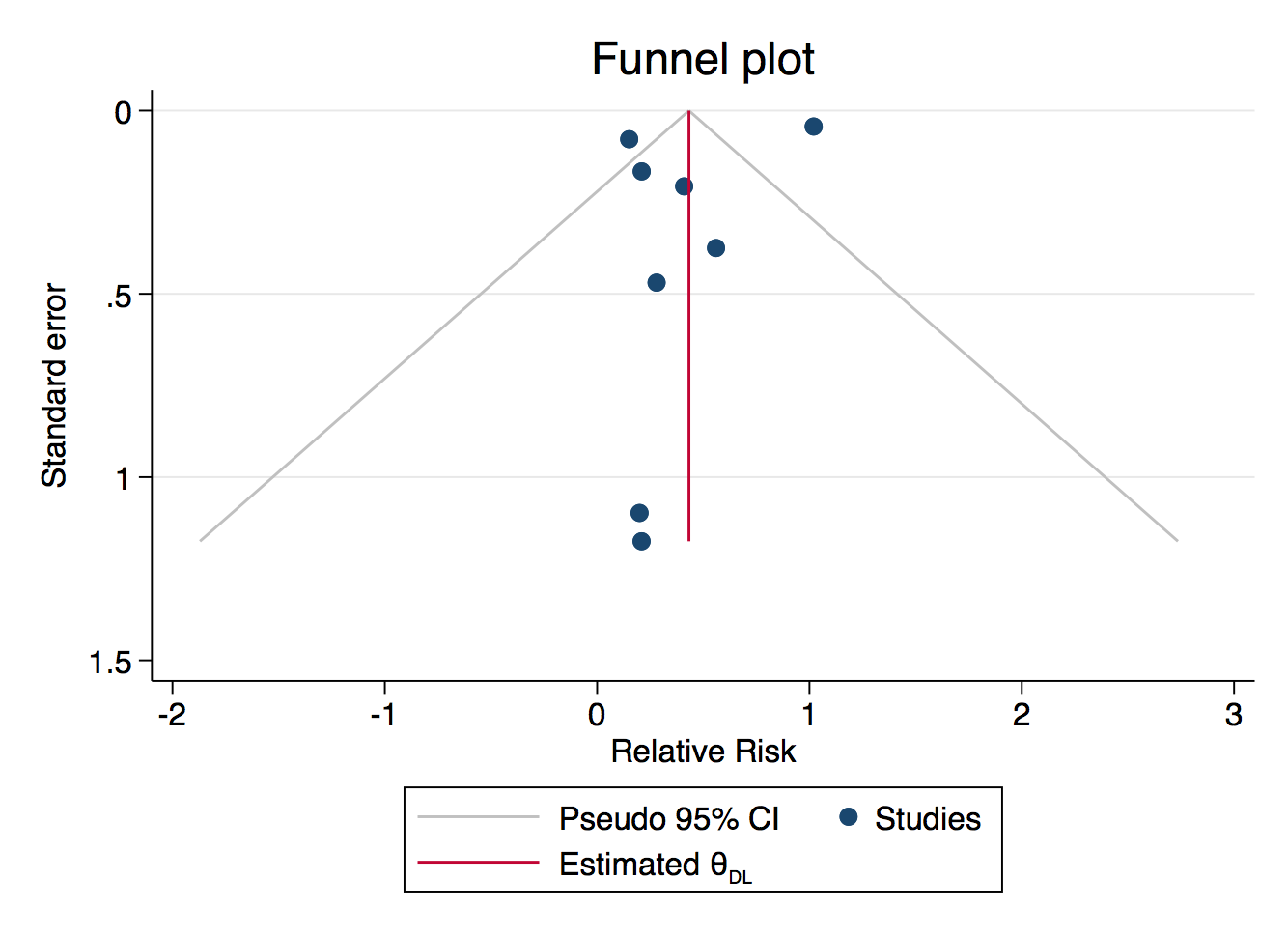

Supplement: S4 Appendix — (DOCX) [file pone.0261358.s005.docx]
